# Upregulated miR-146a expression in peripheral blood mononuclear cells from rheumatoid arthritis patients

**DOI:** 10.1186/ar2493

**Published:** 2008-08-29

**Authors:** Kaleb M Pauley, Minoru Satoh, Annie L Chan, Michael R Bubb, Westley H Reeves, Edward KL Chan

**Affiliations:** 1 Department of Oral Biology, University of Florida, SW Archer Road, Gainesville, Florida 32610, USA; 2 Division of Rheumatology and Clinical Immunology, Department of Medicine, University of Florida, SW Archer Road, Gainesville, Florida 32610, USA; 3 Department of Pathology, Immunology, and Laboratory Medicine, University of Florida, Gainesville, SW Archer Road, Florida 32610, USA

## Abstract

**Introduction:**

MicroRNAs are small noncoding RNA molecules that negatively regulate gene expression via degradation or translational repression of their targeted mRNAs. It is known that aberrant microRNA expression can play important roles in cancer, but the role of microRNAs in autoimmune diseases is only beginning to emerge. In this study, the expression of selected microRNAs is examined in rheumatoid arthritis.

**Methods:**

Total RNA was isolated from peripheral blood mononuclear cells obtained from patients with rheumatoid arthritis, and healthy and disease control individuals, and the expression of miR-146a, miR-155, miR-132, miR-16, and microRNA let-7a was analyzed using quantitative real-time PCR.

**Results:**

Rheumatoid arthritis peripheral blood mononuclear cells exhibited between 1.8-fold and 2.6-fold increases in miR-146a, miR-155, miR-132, and miR-16 expression, whereas let-7a expression was not significantly different compared with healthy control individuals. In addition, two targets of miR-146a, namely tumor necrosis factor receptor-associated factor 6 (TRAF6) and IL-1 receptor-associated kinase 1 (IRAK-1), were similarly expressed between rheumatoid arthritis patients and control individuals, despite increased expression of miR-146a in patients with rheumatoid arthritis. Repression of TRAF6 and/or IRAK-1 in THP-1 cells resulted in up to an 86% reduction in tumor necrosis factor-α production, implicating that normal miR-146a function is critical for the regulation of tumor necrosis factor-α production.

**Conclusions:**

Recent studies have shown that synovial tissue and synovial fibroblasts from patients with rheumatoid arthritis exhibit increased expression of certain microRNAs. Our data thus demonstrate that microRNA expression in rheumatoid arthritis peripheral blood mononuclear cells mimics that of synovial tissue/fibroblasts. The increased microRNA expression in rheumatoid arthritis patients is potentially useful as a marker for disease diagnosis, progression, or treatment efficacy, but this will require confirmation using a large and well defined cohort. Our data also suggest a possible mechanism contributing to rheumatoid arthritis pathogenesis, whereby miR-146a expression is increased but unable to properly function, leading to prolonged tumor necrosis factor-α production in patients with rheumatoid arthritis.

## Introduction

Rheumatoid arthritis (RA) is a systemic autoimmune disorder that is characterized by chronic inflammation of synovial tissue, which results in irreversible joint damage [1]. Inflammatory cytokines, including tumor necrosis factor (TNF)-α and IL-1β, play an important role in RA pathogenesis, and inhibition of these cytokines can ameliorate disease in some patients [2,3].

MicroRNAs (miRNAs) are small noncoding RNA molecules that negatively regulate gene expression at the post-transcriptional level [4,5]. It is predicted that as much as one-third of all mRNAs are targeted for miRNA-mediated regulation [6], and the importance of miRNA regulation is becoming increasingly clear as new roles in critical cellular processes such as apoptosis, differentiation, and the cell cycle are discovered.

The biogenesis and maturation of miRNAs are dependent on two RNase III enzymes, namely Drosha and Dicer. First, miRNAs are transcribed by RNA polymerase II into a long primary miRNA (pri-miRNA) transcript [7,8]. The pri-miRNA is then cleaved by Drosha and its partner protein DGCR8 into an approximately 70-nucleotide precursor miRNA (pre-miRNA) molecule [9-13]. The pre-miRNA is then exported into the cytoplasm via Exportin 5, where it is cleaved into an approximately 21-nucleotide miRNA duplex, similar in structure to small interfering RNA (siRNA) [14,15]. One strand of the miRNA duplex is then loaded into the RNA-induced silencing complex (RISC), where it binds the 3'-untranslated region of its target mRNA, causing the degradation or translational repression of that mRNA [15].

The key components of RISC are the argonaute proteins 1–4 (Ago1–4). Ago2 is known to be the catalytic enzyme of RNA interference and is critical for both miRNA and siRNA function [16,17]. In addition to Ago2, many other proteins are critical for miRNA function, including GW182 and Rck/p54. These proteins, as well as miRNA and siRNA, localize to cytoplasmic foci known as GW or P bodies (here referred to as GWB). Our recent studies have established GWB to be useful biomarkers for siRNA and miRNA activity in cells [18,19]. Our latest study (Pauley KM and coworkers, unpublished data) demonstrated that the number and size of GWB significantly increases concurrently with increased miRNA expression in lipopolysaccharide (LPS)-treated THP-1 cells, implying that GWB can be monitored as biomarkers for miRNA activity.

TNF-α stimulation has been shown to induce the expression of certain miRNAs, including miR-146a and miR-155, in monocytes and macrophages [20,21]. Based on these data and the fact that TNF-α plays an important role in RA pathogenesis, as supported by the development of successful anti-TNF-α therapies, we set out to compare miRNA expression between RA patients and healthy control individuals.

In this study, we obtained peripheral blood mononuclear cells (PBMCs) from RA patients and control individuals and examined the expression of miR-146a, miR-155, miR-132, miR-16, and miRNA let-7a. Most of these miRNAs were chosen for examination based on previous reports linking them to immune stimulation by LPS or TNF-α; miR-16 was selected for its ability to target the 3'-untranslated region of TNF-α [20-22]. miRNA let-7a was chosen as a control. This study is significant because it demonstrates that miRNA expression in RA PBMCs may mimic conditions in synovial tissue and thus enable us to bypass the need for synovial tissue samples, allowing the analysis of larger patient populations.

## Materials and methods

### Patients and control individuals

Sixteen patients (including two samples from a single patient) who fulfilled the American College of Rheumatology classification criteria for RA were included in the study. Their demographic, clinical, and laboratory characteristics are summarized in Table 1. Four disease control individuals, including one with systemic lupus erythematosus, two with Sjögren's syndrome, and one with systemic sclerosis, were included. Nine healthy donors with no history of autoimmune disease were included as control individuals. This study was approved by the University of Florida Institutional Review Board, and written permission was obtained from all who participated in the study.

**Table 1:** Demographic, clinical, and laboratory information of patients

Subject	Sex	Age (years)	Medications	CRP (mg/l)	ESR (mm/hour)
RA Patients					
RA-1a^a^	Male	65	None	4.14^b^	9
RA-1b	Male	65	MTX	11.8	18
RA-2	Female	33	Etanercept, naproxen	25.7	18
RA-3	Female	43	Etanercept	No data	No data
RA-4	Female	45	None	98.4	84
RA-5	Female	55	MTX	4.8	26
RA-6	Female	73	None	2.1	30
RA-7	Female	51	Hydroxychloroquine, MTX, prednisone	No data	15
RA-8	Male	61	Prednisone, MTX, hydroxychloroquine, sulfasalazine	No data	1
RA-9	Male	46	Methylprednisolone, MTX, etanercept	2	9
RA-10	Female	32	Leflunomide	94.2	63
RA-11	Female	50	Etanercept, MTX	No data	No data
RA-12	Male	67	MTX, celecoxib, prednisone	0.6	11
RA-13	Female	55	MTX	No data	17
RA-14	Female	33	Hydroxychloroquine, ibuprofen	2.8	7
RA-15	Female	55	None	168.2	88
RA-16	Female	56	Prednisone, minocycline	No data	No data
Disease control individuals					
SLE1	Female	36	Hydroxychloroquine, prednisone, mycophenolate mofetil		
SjS1	Female	30	Hydroxychloroquine, mycophenolate mofetil		
SjS2	Male	21	Hydroxychloroquine		
SSc1	Female	57	Cyclosporine, hydroxychloroquine, prednisone, mycophenolate mofetil		

^a^Sample collected from RA-1 before and after MTX treatment. ^b^Normal value less than 0.8. The normal CRP is <4.9 mg/l, and the normal ESR is <20 mm/hour for females and <10 mm/hour for males. CRP, C-reactive protein; ESR, erythrocyte sedimentation rate; MTX, methotrexate; RA, rheumatoid arthritis; SLE, systemic lupus erythematosus; SjS, Sjögren's syndrome; SSc, scleroderma.

### PBMC collection and quantitative real-time RT-PCR

Blood samples were collected in EDTA-treated tubes and PBMCs were isolated by standard Ficoll density-gradient centrifugation. PBMCs were washed once in sterile phosphate-buffered saline (PBS) before culture or RNA isolation. Total RNA was isolated from freshly obtained PBMCs using the *mir*Vana miRNA Isolation kit (Ambion, Austin, TX, USA), in accordance with the manufacturer's protocol. RNA concentrations were determined and 10 ng of each RNA sample were used for quantitative real-time RT-PCR (qRT-PCR). miRNA qRT-PCR was performed using the TaqMan MicroRNA Reverse Transcription Kit, TaqMan Universal PCR Master Mix, and TaqMan MicroRNA Assay primers for human miR-146a, miR-155, miR-132, miR-16, and miRNA let-7a (Applied Biosystems, Foster City, CA, USA). mRNA qRT-PCR was performed using the TaqMan High-Capacity cDNA Reverse Transcription Kit, TaqMan Fast PCR Master Mix, and TaqMan mRNA assay primers (Applied Biosystems). All reactions were analyzed using StepOne Real-Time PCR System (Applied Biosystems). The levels of miRNA were normalized to U44 controls, whereas mRNA levels were normalized to 18S RNA. The cycle threshold (Ct) values, corresponding to the PCR cycle number at which fluorescence emission reaches a threshold above baseline emission, were determined and the relative miRNA or mRNA expression was calculated using the 2^-ΔΔCt ^method [23].

### Cell culture and cytokine treatment

THP-1 human monocytes obtained from American Type Culture Collection (Manassas, VA, USA) were cultured in RPMI 1640 medium with 2 mmol/l L-glutamine, 4.5 g/l glucose, 10 mmol/l HEPES, 1.0 mmol/l sodium pyruvate, 0.05 mmol/l 2-mercaptoethanol, and 10% fetal bovine serum. THP-1 cells were seeded at 5 × 10^5 ^cells per well in a six-well plate and treated with 10 ng/ml TNF-α, IFN-γ, IL-12p70, IL-4, IL-10 (BD Biosciences, San Jose, CA, USA), IFN-α, IFN-β (PBL Interferon Source, New Brunswick, NJ, USA), or macrophage colony-stimulating factor (M-CSF; US Biological, Swampscott, MA, USA). Cells were also treated with 25 ng/ml monocyte chemoattractant protein (MCP)-1 (Sigma) in serum-free media. After the designated treatment time had elapsed, cells were harvested and washed once in PBS before analysis.

### Indirect immunofluorescence

THP-1 cells were cytospun onto glass slides at 1,000 rpm for 5 minutes. PBMCs were cultured on glass slides at 37°C for 1 hour. Cells were fixed in 3% paraformaldehyde for 10 minutes and permeabilized in 0.5% Triton X-100 for 5 minutes. GWB were detected in THP-1 cells with a human prototype anti-GWB serum [24] used at 1:6,000 dilution, and in PBMCs with rabbit anti-Rck/p54 antibodies used at 1:500 dilution. TNF receptor-associated factor (TRAF)6 and IL-1 receptor-associated kinase (IRAK)-1 were detected using rabbit anti-TRAF6 (1:50; Santa Cruz Biotechnology, Santa Cruz, CA, USA) and rabbit anti-IRAK-1 (1:50; Santa Cruz Biotechnology). Secondary antibodies used were Alexa Fluor 488 goat anti-human IgG or goat anti-rabbit IgG (1:400) from Molecular Probes (Carlsbad, CA, USA). Slides were mounted using Vectashield Mounting Medium with 4',6-diamidino-2-phenylindole (DAPI; VECTOR Laboratories, Burlingame, CA, USA). Fluorescence images were taken with Zeiss Axiovert 200 M microscope and a Zeiss AxioCam MRm camera using the 20× or 40 × 0.75 NA objectives. Color images were assessed using Adobe Photoshop version 7 (Adobe Systems Inc., San Jose, CA, USA). GWB were counted using Cell-Profiler image analysis software [25].

### siRNA transfection

siRNAs targeting TRAF6 and IRAK-1 were transfected into THP-1 cells using Lipofectamine 2000 (Invitrogen, Carlsbad, CA, USA), in accordance with the manufacturer's instructions. To monitor the transfection efficiency, Cy3-labeled siRNA targeting lamin A/C was transfected into cells in parallel in all transfections, and at least 80% transfection efficiency was achieved. The siRNAs used in this study were all purchased from Applied Biosystems. The sense and antisense strand sequences were as follows: IRAK-1: 5'-GGUUUCGUCACCCAAACAUtt-3' and 5'-AUGUUUGGGUGACGAAACCtg-3'; TRAF6: 5'-GGUUGUUUGCACAAGAUGGtt-3' and 5'-CCAUCUUGUGCAAACAACCtt-3'.

### Multiplex analysis of cytokines

THP-1 cells were transfected as described above and then treated with 1 μg/ml LPS (*Salmonella enterica *serotype minnesota; Sigma, St. Louis, MO, USA) for 24 hours in culture medium. The culture supernatant was then harvested and frozen at -80°C for storage before multiplex analysis. The human cytokine/chemokine LINCOplex premixed kit (LINCO Research, St. Charles, MO, USA) was used in accordance with the manufacturer's protocol in order to detect human MCP-1 and TNF-α quantitatively.

## Results and discussion

### Specific cytokines/chemokines induce GWB in THP-1 cells and human PBMCs

**Figure 1:**
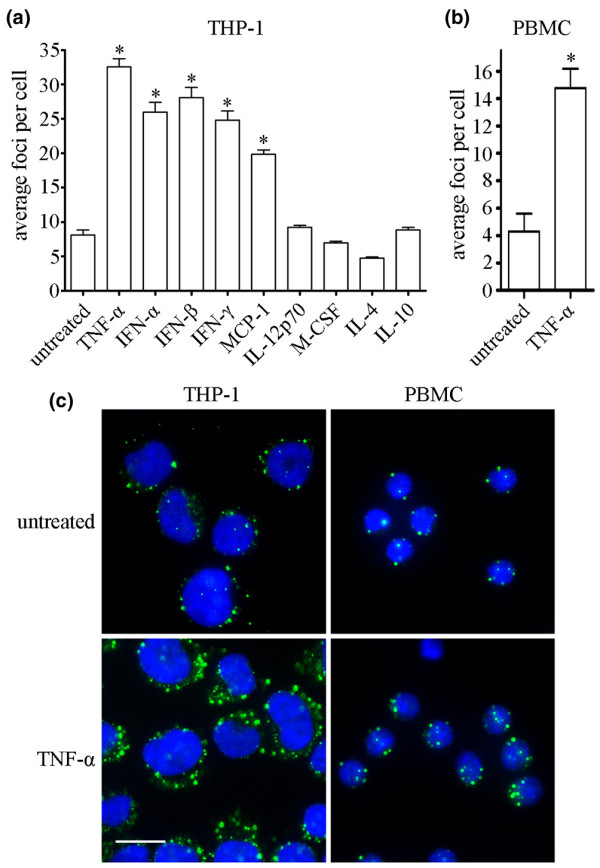
TNF-α treatment results in increased number of GWB in THP-1 and human PBMCs. **(a) **THP-1 cells were treated with 10 ng/ml TNF-α, IFN-α, IFN-β, IFN-γ, IL-12p70, M-CSF, IL-4, IL-10, or 25 ng/ml MCP-1 for 4 hours. IIF was performed using a human anti-GWB serum to detect GWB, and the number of GWB were counted using CellProfiler image analysis software. Average number of GWB per cell and SEM is shown. *P < 0.0001, as determined by one-way analysis of variance. **(b) **Human PBMCs were obtained from a healthy donor and isolated using Ficoll density-gradient centrifugation. The cells were then cultured for 4 hours in the presence of 1 ng/ml TNF-α. GWB were detected by IIF using rabbit anti-Rck/p54 antibodies. Average number of GWB and SEM is shown. *P < 0.0001, as determined by Mann-Whitney test. **(c) **IIF image of THP-1 and PBMCs treated with 10 ng/ml or 1 ng/ml TNF-α for 4 hours, respectively. GWB are shown in green, and nuclei are counterstained with 4',6-diamidino-2-phenylindole (DAPI; blue). Bar = 10 μm. GWP, GW or P bodies; IL, interleukin; IFN, interferon; IIF, indirect immunofluorescence; MCP, macrophage chemoattractant protein; M-CSF, macrophage colony-stimulating factor; PBMC, peripheral blood mononuclear cell; SEM, standard error of the mean; TNF, tumor necrosis factor.

Since our previous work demonstrated that GWB can be used as biomarkers for miRNA activity (Pauley KM, unpublished data), we began to examine a variety of cytokines and chemokines for their ability to stimulate miRNA activity in human monocytic THP-1 cells. THP-1 cells were treated with 10 ng/ml TNF-α, IFN-α, IFN-β, IFN-γ, IL-12p70, M-CSF, IL-4, IL-10, or 25 ng/ml MCP-1 for 4 hours. Indirect immunofluorescence (IIF) was performed using a human anti-GWB serum to detect GWB in the cells. As shown in Figure 1, the proinflammatory cytokines/chemokines TNF-α, IFN-α, IFN-β, IFN-γ, and MCP-1 resulted in a significant increase in the number of GWB per cell compared with untreated cells cultured in parallel (*P *< 0.0001, as determined using one-way analysis of variance). However, IL-12p70, M-CSF, IL-4, and IL-10 had no significant effect on the number of GWB. TNF-α elicited the strongest response in THP-1 cells, with fourfold increase in the average number of GWB per cell (Figure 1a,c). These experiments were repeated at least three times, with reproducible results each time.

Next, we decided to examine the effect of TNF-α stimulation on human PBMC GWB. GWB staining using human PBMCs from a healthy donor, after 4 hours stimulation with TNF-α (1 ng/ml), is shown. Similar to THP-1 cells, the number of GWB per cell increased 3.5-fold after TNF-α stimulation of PBMCs (Figure 1b,c; *P *< 0.0001, as determined by Mann Whitney test). These data indicated that THP-1 cells may be suitable substitutes for human PBMCs in some of the subsequent experiments.

### RA patient PBMCs exhibit increased expression of miR-146a, miR-155, miR-132, and miR-16

In Figure 1 we showed that TNF-α is a potent inducer of GWB and therefore miRNA activity. Our preliminary studies and work from other investigators have confirmed that TNF-α stimulation induces the expression of certain miRNAs, including miR-146a and miR-155 [20,21]. Based on these data and the important role played by TNF-α in RA pathogenesis and therapies, we began to investigate the expression levels of miRNA in RA patients as compared with those in healthy and disease control individuals. PBMCs were obtained from patients (*n* = 17 RA patients and *n *= 4 disease control individuals) and healthy donors (*n* = 9) and isolated by Ficoll density-gradient centrifugation. Initially, RA PBMCs were monitored by IIF for GWB; however, we did not observe an increased number of GWB in RA compared with healthy control individuals (not shown). This discrepancy could be due to limited sensitivity in the quantitation of GWB.

**Figure 2:**
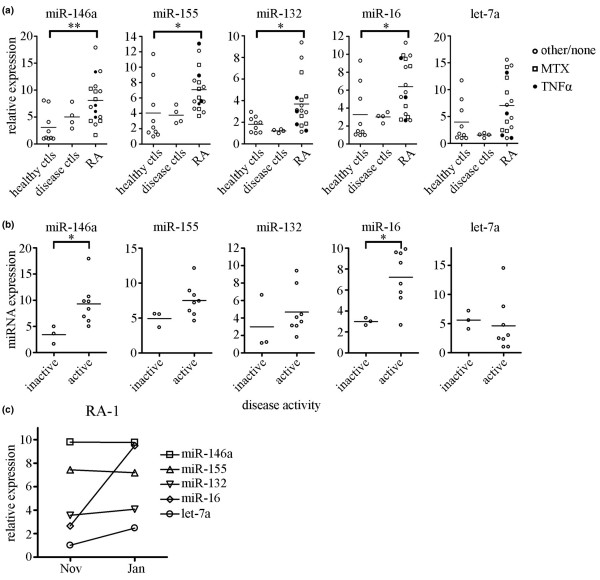
RA patients exhibit aberrant expression of miR-146a, miR-155, miR-132 and miR-16 versus healthy controls. **(a) **RNA was isolated from healthy control individuals (*n* = 9), disease control individuals (*n* = 4), and RA patient (*n* = 17). PBMCs and relative expression levels of miR-146a, miR-155, miR-132, miR-16, and miRNA let-7a were analyzed by qRT-PCR using U44 RNA as an internal control. Average is indicated by bars. *P < 0.05, **P < 0.01, as determined by one-way analysis of variance. For RA patients, closed circles indicate patients undergoing anti-TNF-α therapy at time of sample collection, squares indicate MTX treatment, and open circles indicate other or no treatment. **(b) **Disease activity was determined for patients using CRP and ESR values and correlated with miRNA expression. Normal CRP and ESR values were classified as inactive disease (*n* = 3; patients 9, 12, and 14 in Table 1), and higher than normal CRP or ESR values were classified as active disease (*n* = 8; patients 1a, 1b, 2, 4, 5, 6, 10, and 15 in Table 1). Those patients with no or incomplete data for CRP/ESR values were omitted. *P < 0.05, as determined by t-test. **(c) **PBMCs were collected from patient RA-1 before (November 2007) and after (January 2008) MTX treatment and miRNA expression was examined using qRT-PCR. miRNA expression is largely consistent over time, with the exception of increased miR-16 expression. CRP, C-reactive protein; ESR, erythrocyte sedimentation rate; miRNA, microRNA; MTX, methotrexate; PBMC, peripheral blood mononuclear cell; qRT-PCR, quantitative real-time RT-PCR; RA, rheumatoid arthritis; RT-PCR, reverse transcription polymerase chain reaction; TNF, tumor necrosis factor.

As shown in Figure 2a, the average relative expression levels of miR-146a, miR-155, miR-132, and miR-16 were 2.6-, 1.8-, 2.0-, and 1.9-fold, respectively, higher for RA patients than for healthy control individuals (*P *< 0.01 for miR-146a and *P *< 0.05 for miR-155, miR-132, and miR-16, as determined by one-way analysis of variance). The expression of miRNA let-7a was not significantly different between RA patients and healthy control individuals (Figure 2a). Disease control miRNA expression resembled that in healthy control individuals.

To examine the relationship between RA disease activity and miRNA expression levels, patients were classified into inactive/remission and active patients, based on C-reactive protein (CRP) and erythrocyte sedimentation rate (ESR) values. Three patients with normal CRP and ESR (Table 1) were classified as inactive, whereas eight patients with elevated CRP and/or ESR (Table 1) were classified as active (Figure 2b). Those patients with incomplete or no available data were omitted (Table 1). miRNA expression levels were compared between the groups. Interestingly, high miR-146a and miR-16 expression levels appeared to correlate with active disease, whereas low expression level correlated with inactive disease (Figure 2b; *P *< 0.05, as determined by t-test). These data indicates that miR-146a and miR-16 expression levels may be a useful marker of RA disease activity. Further studies involving a larger patient cohort are needed to determine fully whether monitoring miRNA expression as a marker for disease activity can improve upon CRP or ESR measurements.

Figure 2c shows the miRNA expression levels in two samples from a single RA patient collected over a 2-month interval, during which time this patient's CRP and ESR values increased despite methotrexate treatment. The miRNA levels of this patient were largely unchanged over the 2-month interval, remaining elevated compared with those in healthy control individuals. This indicates that the elevated miRNA expression in this patient may reflect the patient's lack of improvement, as indicated by the increased CRP and ESR values. In this patient, miR-146a, miR-155, and miR-132 expression levels were stable over this time period, whereas the expression levels of miR-16 and miRNA let-7a increased by approximately 3.5-fold and 2.4-fold, respectively. A larger patient population must be examined in order to determine whether miRNA expression levels may be indicative of treatment efficacy.

To further analyze the increased miRNA expression exhibited by these RA patients, we compared miR-146a, miR-155, and miR-132 expression levels with patient clinical and demographic data (Table 1) and found no significant trends or correlations between high expression levels and age, race, or medications. Patients receiving no medications at the time of miRNA analysis exhibited the same trend toward elevated miRNA expression, indicating that treatment with medications is not responsible for the increased miRNA expression in RA patients.

Recently, two reports [26,27] showed increased miR-146 and miR-155 expression levels in RA synovial tissue and fibroblasts. Stanczyk and coworkers [27] reported a fourfold increase in miR-146a expression and a twofold increase in miR-155 expression in RA synovial fibroblasts compared with osteoarthritis synovial fibroblasts. They also demonstrated that miR-155 expression can repress the induction of matrix metalloproteinases 3 and 1, indicating that miR-155 may be involved in modulating the destructive properties of RA synovial fibroblasts. However, in that report, miR-155 expression from RA PBMCs was not significantly different from that in control PBMCs. This discrepancy could be due to differences in experimental techniques or patient populations. Nakasa and colleagues [26] also reported an approximately fourfold increase in miR-146a expression in RA synovial tissue. Our data demonstrate that RA patient PBMCs exhibit elevated miRNA expression in a similar manner to RA synovial tissue, with a 2.6-fold increase in miR-146a expression and a 1.8-fold increase in miR-155 expression. Because of the invasiveness involved in collecting samples, monitoring miRNA expression in RA synovial tissue is, in most cases, limited to extremely severe disease in patients undergoing joint surgery or replacement. Because blood collection is not invasive, this allows for easy sample collection over time, which is a distinct advantage when monitoring disease activity and treatment efficacy.

### Monocyte/macrophage population of RA PBMCs exhibits increased miRNA expression

Because PBMCs are composed of a mixed cell population, the two main components of which are monocytes/macrophages and lymphocytes, we wished to determine which cell population in RA patients exhibits increased miRNA expression. PBMCs were isolated from RA patients (*n* = 2) and incubated in tissue culture dishes at 37°C for 1 hour. The monocyte/macrophage population adhered to the dish, whereas lymphocytes remained in suspension. The adherent cells were washed five times with sterile PBS, and the nonadherent cells were collected and washed with sterile PBS. The purity of the adherent population was approximately 80%, as determined by microscopy. RNA was isolated from the cells, miRNA expression was analyzed by qRT-PCR, and the data were normalized within the total group of patient and control samples.

**Figure 3:**
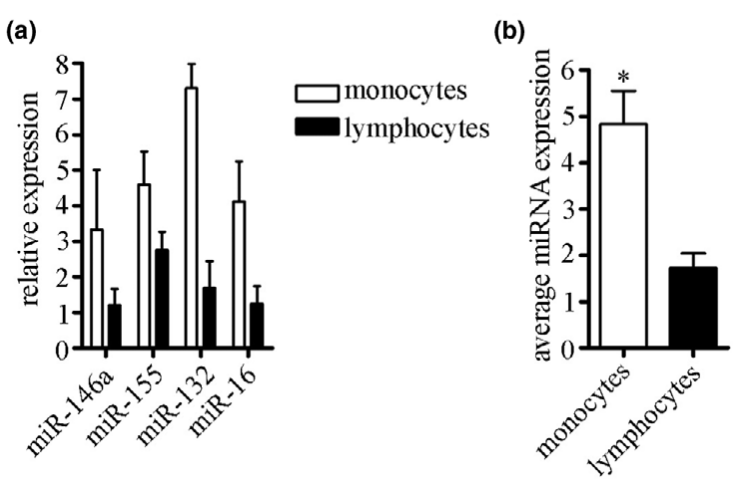
Monocyte/macrophage fraction of PBMCs exhibit increased miRNA expression compared with lymphocyte fraction. PBMCs were collected from RA patients and separated into monocyte/macrophage and lymphocyte populations by allowing the monocytes/macrophages to adhere to a tissue culture dish. **(a) **miRNA expression was examined using qRT-PCR. SEM is shown (*n* = 2 patients). **(b) **Average expression levels of miR-146a, miR-155, miR-132, and miR-16 are shown for monocyte and lymphocyte populations for two RA patients. *P < 0.02, as determined by Mann-Whitney test. SEM is shown. PBMC, peripheral blood mononuclear cell; qRT-PCR, quantitative real-time RT-PCR; RA, rheumatoid arthritis; RT-PCR, reverse transcription polymerase chain reaction; SEM, standard error of the mean.

The expression levels of miR-146a, miR-155, miR-132, and miR-16 were 2.8-, 1.6-, 4.2-, and 3.4-fold higher, respectively, in monocytes than in lymphocytes (Figure 3a). Let-7a expression was similar between monocytes and lymphocytes (not shown). Figure 3b shows the average expression (miR-146a, miR-155, miR-132, and miR-16 combined) for the monocyte and lymphocyte populations of two RA patients (*P *< 0.02, as determined by Mann-Whitney test). These findings suggest that monocytes/macrophages contribute to the increased miRNA expression observed in RA patients more than lymphocytes, but further studies must be performed to confirm this observation.

### TRAF6 and IRAK-1 expression is similar between RA patients and control individuals

**Figure 4:**
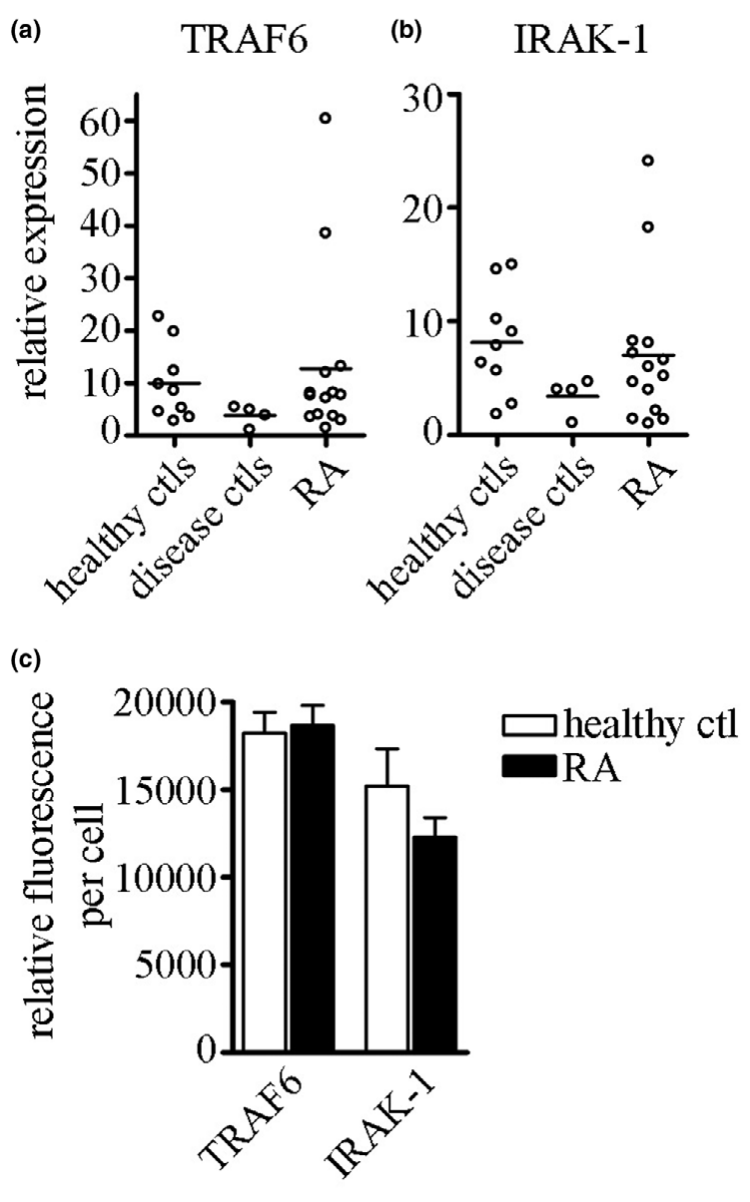
TRAF6 and IRAK-1 expression levels are similar between RA patients, healthy controls, and disease controls. RNA was isolated from PBMCs from healthy control individuals (*n *= 9), disease control individuals (*n* = 4) and RA patients (*n* = 14), and mRNA expression levels of **(a) **TRAF6 and **(b) **IRAK-1 were analyzed using qRT-PCR. **(c) **PBMCs isolated from a healthy control individual and RA patient were incubated on glass slides for 1 hour at 37°C. The adhered cells were fixed and permeabilized in 3% paraformaldehyde and 0.5% Triton X-100, respectively. Protein levels of TRAF6 and IRAK-1 were analyzed by immunofluorescence using rabbit anti-TRAF6 and anti-IRAK-1 antibodies, and relative fluorescence was determined using Image J analysis software. SEM is shown; *n *> 20 cells. IRAK, IL-1 receptor-associated kinase; PBMC, peripheral blood mononuclear cell; qRT-PCR, quantitative real-time RT-PCR; RA, rheumatoid arthritis; RT-PCR, reverse transcription polymerase chain reaction; SEM, standard error of the mean; TRAF, tumor necrosis factor receptor-associated factor.

Because most of the RA patients exhibited increased expression of miR-146a compared with healthy and disease control individuals, we decided to examine the expression of two confirmed targets of miR-146a, namely TRAF6 and IRAK-1 [21]. TRAF6 and IRAK-1 mRNA expression levels were analyzed by qRT-PCR (Figure 4a,b). RA patients exhibited increased miR-146a production compared with control individuals, and we therefore expected to observe decreased TRAF6 and/or IRAK-1 expression in RA patients as compared with control individuals. However, TRAF6 and IRAK-1 mRNA expression levels were very similar between RA patients and control individuals, and overall the mRNA levels of TRAF6 and IRAK-1 did not exhibit the same degree of variability between patients that we observed with miRNA expression. This may indicate that TRAF6 and IRAK-1 transcripts are under other levels of control. To confirm this discrepancy, we analyzed TRAF6 and IRAK-1 protein levels by IIF in one healthy control individual and one RA patient whose miR-146a level was increased (Figure 2a). PBMCs were processed for IIF as previously described and were stained for TRAF6 and IRAK-1. Image J software was used to quantify the relative level of fluorescence for at least 20 cells. As shown in Figure 4c, there was no significant difference in TRAF6 or IRAK-1 protein levels between the RA patient and healthy control individual, which is consistent with the mRNA analysis.

**Figure 5:**
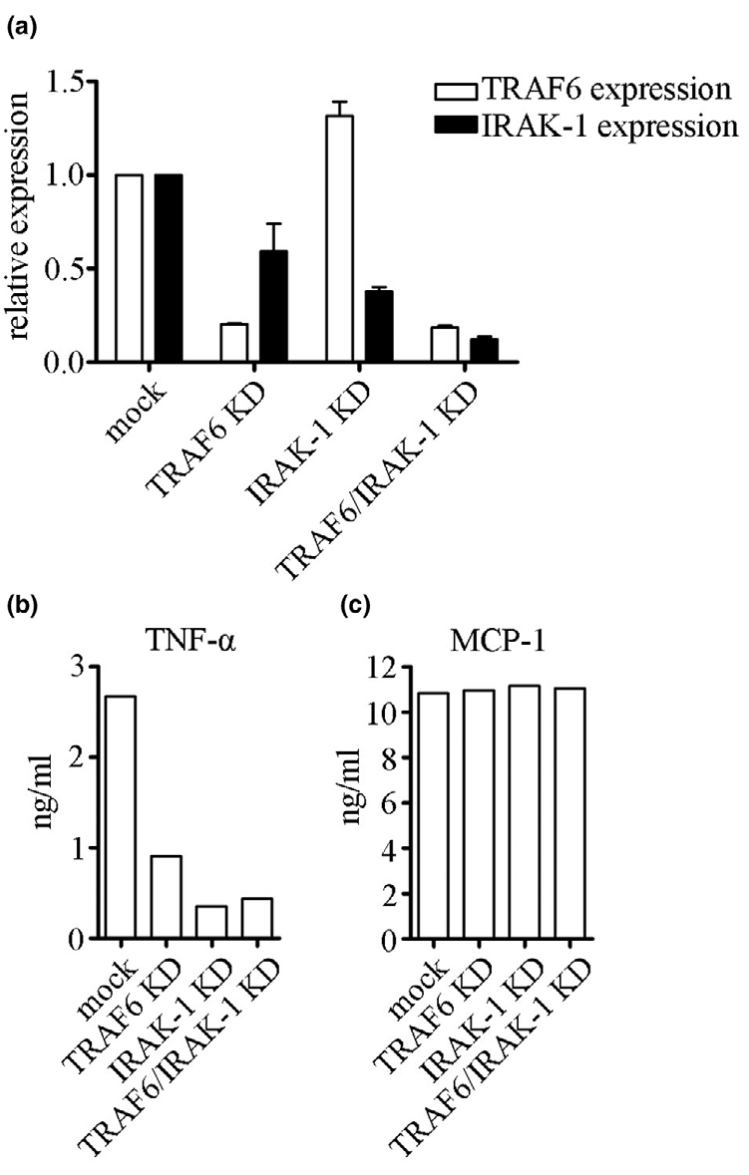
Knockdown of TRAF6 and/or IRAK-1 results in decreased TNF-α production in THP-1 cells. THP-1 cells were transfected with siRNA targeting TRAF6 and/or IRAK-1. **(a) **48 hours after transfection, mRNA levels of TRAF6 and IRAK-1 were analyzed by qRT-PCR and normalized to mock transfected cells. SEM shown (*n* = 2). After knockdown of TRAF6 and/or IRAK-1 was confirmed by qRT-PCR, cells were treated with 1 μg/ml LPS for 24 hours and culture supernatants were collected. Multiplex assay was used to quantitatively detect **(b) **TNF-α and **(c) **MCP-1. IRAK, IL-1 receptor-associated kinase; LPS, lipopolysaccharide; MCP, macrophage chemoattractant protein; PBMC, peripheral blood mononuclear cell; qRT-PCR, quantitative real-time RT-PCR; RT-PCR, reverse transcription polymerase chain reaction; TNF, tumor necrosis factor; TRAF, tumor necrosis factor receptor-associated factor.

It is interesting to speculate that this lack of regulation of TRAF6/IRAK-1 by miR-146a could play a role in RA pathogenesis, especially because it has been reported that inhibition of IRAK-1 using antisense oligonucleotides results in decreased LPS-induced cytokine production [28], and our preliminary data have shown that transfection of miR-146a into THP-1 monocytes results in knockdown of TRAF6 and IRAK-1 expression and inflammatory cytokine production (Pauley KM and coworkers, unpublished data). To investigate this possibility further, we transfected siRNA targeting TRAF6 and/or IRAK-1 into THP-1 cells. The knockdown efficiency was determined by analyzing TRAF6 and IRAK-1 mRNA levels by qRT-PCR, and at least 80% and 60% knockdown was achieved for TRAF6 and IRAK-1, respectively (Figure 5a). Two days after transfection, knockdown and control cells were treated with 1 μg/ml LPS for 24 hours. Culture supernatants were collected and cytokines/chemokines were quantitatively detected using a human cytokine multiplex assay. TNF-α production was drastically reduced in the TRAF6 and/or IRAK-1 deficient cells compared with mock transfected cells (Figure 5b), whereas MCP-1 production was not affected by TRAF6 or IRAK-1 knockdown (Figure 5c).

These data demonstrate that TRAF6 and IRAK-1 are required for the production of TNF-α in THP-1 cells. Taken together, it is reasonable to hypothesize that the absence of TRAF6/IRAK-1 regulation by miR-146a in RA patients could contribute to the prolonged production of TNF-α that many of these patients exhibit. Furthermore, it would be interesting to investigate the expression patterns of miR-146a, TRAF6, and IRAK-1 in RA patients who are responsive to anti-TNF-α therapy versus those who are not responsive. Clearly, further studies are needed to elucidate the role played by miR-146a regulation in RA pathogenesis and the mechanism by which TRAF6/IRAK-1 escape miR-146a regulation.

## Conclusion

In summary, this study demonstrates that PBMCs from RA patients exhibit statistically significant increased expression levels of miR-146a, miR-155, miR-132, and miR-16 compared with healthy and disease control individuals. Furthermore, we demonstrated that high levels of miR-146a and miR-16 expression correlate with active disease, whereas low expression levels correlate with inactive disease. Although miR-146a expression is increased in RA patients, levels of the two established miR-146a targets TRAF6 and IRAK-1 in RA patients are similar to those in control individuals. We also show that TRAF6 and IRAK-1 regulation is important for TNF-α production in THP-1 cells.

Normally, stimuli such as LPS or TNF-α will induce expression of miR-146a, miR-155, and miR-132 in a nuclear factor-κB dependent manner [21]. In the case of miR-146a, this will lead to negative regulation of TRAF6 and IRAK-1, which in turn will decrease the production of proinflammatory cytokines/chemokines, including TNF-α. Thus, the function of miR-146a, at least in part, is to control the extent of the stimulation, such that the production of some of these proinflammatory cytokines/chemokines will not continue for an extended period of time. However, it is interesting to speculate that defective negative regulation of TRAF6 or IRAK-1 by the increased miR-146a in RA patients is the cause of prolonged TNF-α production.

Although it is very exciting that two independent studies have shown increased miRNA expression in RA synovial tissue, analyzing miRNA expression in patient PBMCs presents a distinct advantage over analyzing synovial tissue samples. Collection of PBMCs is noninvasive, and samples can be collected from patients ranging in disease severity from early onset to more severe, whereas synovial tissue collection is biased toward patients with severe degenerative disease. With further validations and studies conducted in larger patient populations, monitoring of selected miRNAs could prove to be a valuable addition to RA diagnostics, or monitoring disease progression or treatment efficacy. The underlying mechanisms resulting in increased miRNA expression and inability of miR-146a to regulate its targets need to be elucidated, and these mechanisms may be potential targets for the development of new RA therapies.

## Abbreviations

CRP: C-reactive protein; ESR: erythrocyte sedimentation rate; GWP: GW or P bodies; IL: interleukin; IFN: interferon; IIF: indirect immunofluorescence; IRAK: IL-1 receptor-associated kinase; LPS: lipopolysaccharide; MCP: monocyte chemoattractant protein; M-CSF: macrophage colony-stimulating factor; miRNA: microRNA; PBMC: peripheral blood mononuclear cell; qRT-PCR: quantitative real-time RT-PCR; RA: rheumatoid arthritis; RISC: RNA-induced silencing complex; RT-PCR: reverse transcription polymerase chain reaction; siRNA: small interfering RNA; TNF: tumor necrosis factor; TRAF: tumor necrosis factor receptor-associated factor.

## Competing interests

The authors declare that they have no competing interests.

## Authors' contributions

KMP designed and conducted all experiments and drafted the manuscript. MS assisted with statistical evaluations, provided clinical insights, and edited the manuscript. ALC collected patient samples. MRB and WHR recruited study subjects, and provided clinical insights and advice. EKLC conceived of the study, assisted in designing the study, and edited the manuscript. All authors read and approved the final manuscript.
